# Filling Kinetic Gaps: Dynamic Modeling of Metabolism Where Detailed Kinetic Information Is Lacking

**DOI:** 10.1371/journal.pone.0004967

**Published:** 2009-03-23

**Authors:** Osbaldo Resendis-Antonio

**Affiliations:** Center for Genomic Sciences-UNAM, Cuernavaca Morelos, Mexico; German Cancer Research Center, Germany

## Abstract

**Background:**

Integrative analysis between dynamical modeling of metabolic networks and data obtained from high throughput technology represents a worthy effort toward a holistic understanding of the link among phenotype and dynamical response. Even though the theoretical foundation for modeling metabolic network has been extensively treated elsewhere, the lack of kinetic information has limited the analysis in most of the cases. To overcome this constraint, we present and illustrate a new statistical approach that has two purposes: integrate high throughput data and survey the general dynamical mechanisms emerging for a slightly perturbed metabolic network.

**Methodology/Principal Findings:**

This paper presents a statistic framework capable to study how and how fast the metabolites participating in a perturbed metabolic network reach a steady-state. Instead of requiring accurate kinetic information, this approach uses high throughput metabolome technology to define a feasible *kinetic* library, which constitutes the base for identifying, statistical and dynamical properties during the relaxation. For the sake of illustration we have applied this approach to the human Red blood cell metabolism (hRBC) and its capacity to predict temporal phenomena was evaluated. Remarkable, the main dynamical properties obtained from a detailed kinetic model in hRBC were recovered by our statistical approach. Furthermore, robust properties in time scale and metabolite organization were identify and one concluded that they are a consequence of the combined performance of redundancies and variability in metabolite participation.

**Conclusions/Significance:**

In this work we present an approach that integrates high throughput metabolome data to define the dynamic behavior of a slightly perturbed metabolic network where kinetic information is lacking. Having information of metabolite concentrations at steady-state, this method has significant relevance due its potential scope to analyze others genome scale metabolic reconstructions. Thus, I expect this approach will significantly contribute to explore the relationship between dynamic and physiology in other metabolic reconstructions, particularly those whose kinetic information is practically nulls. For instances, I envisage that this approach can be useful in genomic medicine or pharmacogenomics, where the estimation of time scales and the identification of metabolite organization may be crucial to characterize and identify (dis)functional stages.

## Introduction

Constraints-based modeling represents a paradigm in systems biology with a broad scope of applications ranging from bioengineering to cellular evolution [1], [2], [3], [4], [5], [6], [7], [8], [9]. Briefly, constraints-based models is a bottom-up scheme that use the successive imposition of constraints (such as mass conservation, fundamental thermodynamic and enzymatic capacity) to delimit the functional space of a metabolic network. Mathematically, functional space is entirely obtained by the stoichiometric matrix when one assume that all metabolic fluxes do not change in time, it means all reactions conforming the network obey the steady-state condition.

Parallel to these *in silico* modeling, the data supplied from high throughput technologies has triggered the development of deductive top-down procedures, in order to complement and verify biological predictions obtained from constraints-based models [10], [11].

Even though constraints-based *in silico* models have provided a successful method for accomplishing the integrative task between high throughput data and genome scale models, the steady-state assumption may oversimplify cellular behavior such that its description is valid only at certain time scales. In order to deal with metabolic mechanism away from a steady-state, it is imperative to develop new genome scale *in silico* models capable to provide a temporal description of the cell activity and relay it with its physiological behavior [12], [13], [14].

For instance, a paradigm linking dynamic and physiological behavior is clearly manifested in human red blood cell metabolism (hRBC) [15], [16]. Thus, modeling hRBC metabolism has permitted us to explore the dynamic effects produced by the lack of certain enzymatic activity, for example glucose 6-Phosphate dehydrogenase, and to correlate this metabolite deficiency with enzymopathies at various clinical stages [15], [17], [18]. Unfortunately, detailed dynamical studies, such as those carried out for hRBC cannot be extended to other cell metabolisms mainly because of the lack of specific kinetic information. Even though a number of databases storing kinetic data are currently being assembled [19], [20], [21], this fundamental constraint reveals the need to develop novel approaches for estimating kinetic parameters and explore dynamic properties in genome scale metabolic reconstructions [9], [14], [22], [23], [24], [25].

**Figure 1:**
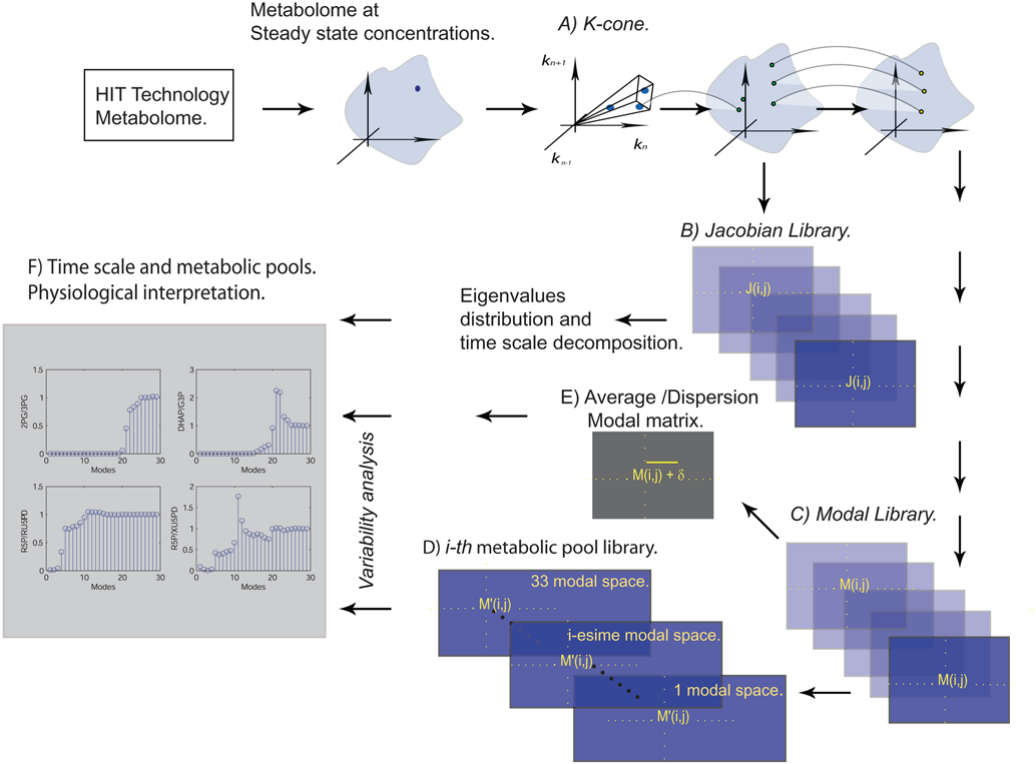
General overview. A) Based on metabolome data and reconstructed metabolic network, we obtain the feasible set of kinetic parameters, *k-cone*. A point in this space represents a vector whose dimension is given by the number of reactions in the metabolic network. B) To explore the relationship between physiology and dynamic behavior for a perturbed metabolic network where kinetic parameters is lacking, we construct a *Jacobian* library taking into account the *k-cone* space. A point in *Jacobian* library represents a square matrix with dimension determined by the number of metabolites. C) In turn, for each *Jacobian* we obtain a *modal* matrix, we called this new space the *Modal* library. D) In order to analyze the variability for each of the modes along the ensemble we define *i-th metabolic pool* library, a subspace of the entire *modal* library. E) We calculate the average properties, the dispersion for the time scales and the *metabolic pools* generated along the library. F) The statistical analysis of the time scales and the metabolic organization are interrelated to infer metabolites with potential physiological meaning.

In this work I suggest a statistical framework to analyze dynamical properties of a metabolic network when its metabolite concentrations are slightly perturbed around a steady-state. To overcome the lack of kinetic parameters, this approach uses high throughput metabolome data for obtaining a *kinetic* library conformed by all the kinetic parameters which dynamically ensure the existence of a steady-state solution. Subsequently, through this kinetic space, one constructs a library of dynamical models, all of them characterized by the same metabolic network but predicting dynamic behavior with different kinetic parameters. As this paper suggests, a statistical analysis applied over the library of dynamical models allows us to survey general properties even in the absence of accurate kinetic information. The library of dynamical models constitutes a fundamental space required to explore two immediately questions: how and how fast a metabolic network reaches its steady-state after a slightly external perturbation has occurred. The workflow of the method is such that it integrates three main components: metabolome data [26], [27], the stoichiometric matrix (holding the metabolic biochemical reactions in the organism) and the classical theory of modal analysis [28]. A schematic overview of the approach is depicted in Figure 1.

The solution space of feasible kinetic parameters, defined as the *k-cone*, has been calculated considering three requirements: 1) All biochemical reactions obey law mass action, 2) Steady-state metabolite concentrations are known quantities and potentially obtained from metabolome data [29], and 3) All kinetic parameters defining the *k-cone* are such that they dynamically ensure the existence of a steady-state solution, see methods section.

Having defined *k-cone*, the statistical nature of this framework emerges when one constructs two additional libraries, *Jacobian* and *Modal* [28], [30], whose components are matrices with information referring how and how fast the system reaches a steady-state.

More specifically, given certain kinetic parameters, *Jacobian* matrix defines the times scales and the *Modal* matrix specify how metabolites organize to reach its steady-state. As this paper supports, statistical analysis accomplished in both libraries can potentially guide us to identify: 1) robust dynamical properties inherent to the biological systems, 2) scrutinize how dynamic properties depend on specific kinetic parameters, and 3) potentially associate dynamical with functional behavior in cell.

In order to illustrate the scope of our method we will apply this to the Human Red Blood Cell (hRBC) metabolic network. In this paper, hRBC metabolic reconstruction is conformed by 33 metabolites participating in 68 biochemical and transport reactions, *see Table S1* and *Table S2* in *supporting information*. Overall, I believe this approach, schematically represented in Figure 1, can act as a guide for exploring the relationship between dynamics and physiology behavior in metabolic networks where the kinetics is partially or completely unknown.

## Results

### Building kinetic and dynamical libraries

*K-cone* analysis is a formalism useful to determine a space containing all candidate values for kinetic parameters of a metabolic network [29]. *K-cone* space for hRBC metabolic network was obtained taking into account the stoichiometric matrix and a set of metabolite concentrations defining a steady-state condition [29]. As described in *method section*, two assumptions were applied during the analysis. Firstly, the concentration of all the metabolites at a steady-state was supposed to be known, *see Table S1 in supporting information*. This assumption particularly encourages to use accurate data obtained from high throughput metabolome technology [26]. Secondly, we have considered that all metabolic reactions are governed by the law of mass action. Even though this latter assumption may limit the practical kinetic scope, this method can immediately be extended to include crowding effects through applying a generalized mass action [29]. These assumption and mass conservation can be combined to obtain Equation 2, see method section, whose solution is conformed by a myriad of vectors that inside a multidimensional cone, the called *k-cone* space, see Figure 1 A.

Thus, a point inside this space represents a vector with 68 entries, each one defining a kinetic parameter corresponding for the 68 biochemical reactions included in hRBC metabolic network. Having identified *k-cone* space, one proceeds to construct a *kinetic* library simply by randomly selecting some points inside *k-cone* space. Mathematically, random sampling procedure was accomplished by applying an Artificial Center Hit and Run algorithm [31], [32] selecting 19000 points and storing those (12586) that ensure the existence of a steady-state coinciding with the known steady-state metabolite level, *see method section*. As a result, numerical distributions associated with the kinetic parameter for each metabolic reaction were obtained. The resulting ranges of values for each biochemical reaction are reported in *Table S3 at supporting information*.

Once *kinetic* library was obtained, one can proceeded to survey how and how fast the metabolites reaches the steady-state after a slightly perturbation. In general, biochemical reactions integrating metabolic networks occurs over a broad spectrum of time scales, such that some of them are faster than others depending on its kinetic parameters and presumably on its physiology relevance [33]. For a given set of kinetic parameters, time scale decomposition for hRBC metabolic network was classically obtained through the *Jacobian* matrix, *see methods section*. In essence the entries in this matrix indicate how fast a metabolic flux changes when the concentration of certain metabolite changes too. Subsequently, a library of feasible time scales was constructed by evaluating each component on *kinetic* library in the *Jacobian* matrix, and proceeding as described in *methods section*, the time scale proper for each set of kinetic parameters were calculated. The resulting distribution of hierarchical time scale was gathered for constructing a *hierarchical time scale* library. Furthermore, in order to reveal how the metabolites organize between them for recovering its steady-state, we used a *modal* matrix analysis which was directly obtained from the *Jacobian* library, see method section and Figure 1.

*Modal* matrix analysis is formalism for identifying the metabolites that coordinately moves at a specific time scale, called metabolic pools. According to modal theory, for each one *Jacobian* matrix (*J*) it is possible to find a *Modal* matrix (*M*) whose rows supply information of the metabolic pools at each time scale, *see methods section*. Thus, as we did before, a *modal* library was constructed by storing all *Modal* matrices obtained for each element inside the *Jacobian* library, see methods and Figure 1.

Based on a statistical analysis applied to the *hierarchical time scales* and *modal* library, next section is devoted to show that one can tackle two interesting issues: to uncover global properties in dynamic metabolic networks and to survey the proper metabolic organization required to induce adequate physiological functions. An overview of the methodology and the relationship between its libraries is schematically represented in Figure 1.

### Robustness in time scale decomposition

**Figure 2:**
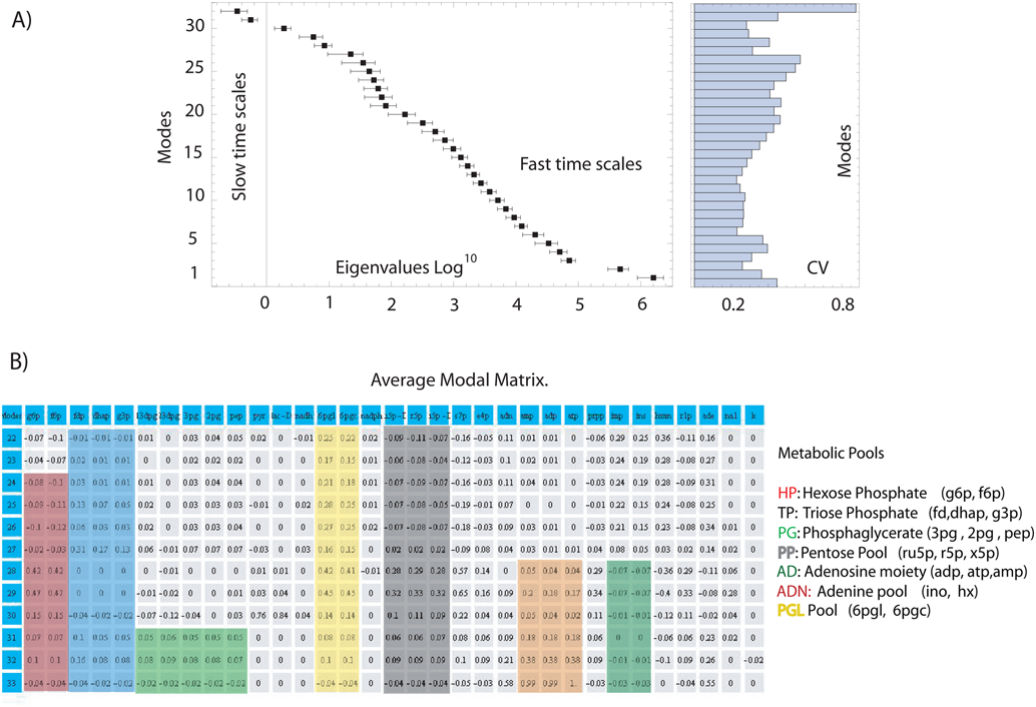
Time scale and metabolic pools for human Red Blood Cell metabolism. Figure (A) shows the distribution of the eigenvalues (log_10_) obtained from the *Jacobian* library. Coefficient of variation (CV) calculated for the resulting eigenvalues distribution is depicted at the right side of the plot. Figure (B) depicts the average modal matrix. At slow times scales it is possible to identify metabolic pools that correlate with physiological functions [15]. The metabolites are denoted by: 13dpg ,1,3-Diphosphoglycerate; 23dpg, 2,3-Diphosphoglycerate; 2pg, 2 Phosphoglycerate; 3pg, 3-Phosphoglycerate; and, Adenine; adp, Adenosine diphosphate; amp, Adenosine monophosphate; atp, Adenosine triphosphate; dhap, Dihydroxyacetone phosphate; e4p, Erythrose-4-phosphate; f6p, Fructose-6-phosphate; fdp, Fructose-1,6-diphosphate; g3p, Glyceraldehyde 3-phosphate; g6p, D-glucose 6-phosphate; hxan, Hypoxanthine; imp, Inosine monophosphate; ins, Inosine; k, Potassium; lac-D, D-lactate; na1, Sodium; nadh, Nicotinamide adenine dinucleotide; pep, Phosphoenolpyruvate; prpp, 5-Phospho-alpha-D-ribose-1-diphosphate; pyr, Pyruvate; r1p, alpha-D-Ribose 1-phosphate; r5p, alpha-D-Ribose 5-phosphate; ru5p-D, D-Ribulose 5-phosphate; s7p, Sedoheptulose 7-phosphate; xu5p-D, D-Xylulose 5-phosphate.

**Figure 3:**
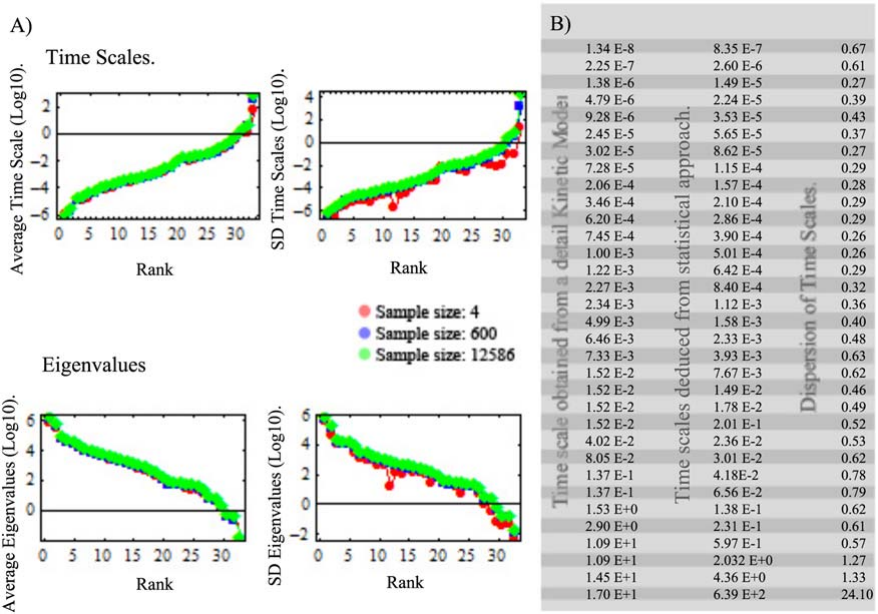
Convergence and accuracy of the algorithm. Figure (A), upper panel, shows the average time scale and the corresponding standard deviation obtained for three different kinetic sample sizes. Equivalently, the same procedure was accomplished for the eigenvalues, see Figure (A) at bottom panel. Panel (B) shows the range of time scale deduced from the statistic method (with 12586 points in *k-cone*) and the reported by a detail kinetic model [15].

To uncover the role that kinetic parameters have onto time scale profiles, a statistical analysis over the *hierarchical time scale library* was accomplished. Thus, proceeding as described in *methods section* (Equation 6), the average and the standard deviation for the inverse of each time scale obtained from *Jacobian* library were calculated and plotted in Figure 2A Two relevant results emerge from this result. Firstly, inverse of times scale reveals a low dispersion respect to the average, thus indicating the presence of robust statistical properties on kinetic parameters, see Figure 2A, left panel. Furthermore, repeating the same statistical analysis now directly over the profile of time scales, one obtain that robustness prevails for all time scales, except for the last three slowest ones, indicating its sensitivity to kinetic parameters, *see Figure S1 in supporting information*. Robustness is a property that has been reported in a variety of biological systems [34], [35]. In this paper, we present evidence to indicate that time scale associated with a perturbed metabolic networks is statistical robust to kinetic parameters for most time scales, and only in the case of slow time regimens they can have a considerable influence on dynamics. To evaluate the robustness of this result and heuristically evaluate the dispersion intrinsic of the network and the coming from sampling artifact, I have explored how the statistical properties of the eigenvalues and time scales vary at different *k-cone* sample sizes. Figure 3A shows the average and standard deviation (both in log_10_ scale) for the eigenvalues and time scales obtained when one select 4, 600 and 12586 points in *k-cone* space. Particularly, we note that in all cases the average and dispersion converge according the number of sample size increase. Notably, this approach allows to identify time scales that are closely similar to those obtained by using a detail kinetic model for hRBC [15], [18], see Figure 3B.

In summary, fast and intermediate time scales tend to be statistically robust to kinetic parameter variations. Robustness, in this case, may be a consequence that the metabolic network compensates an altered kinetic property by using an equivalent functional metabolic pathway. Conversely, the last three slow time scales are sensitive to variations in kinetic parameters and it may be caused by a reducing number of alternative pathways to replace the effects of kinetic variations. Based in this hypothesis, we postulate that robustness in time scale can be correlated with the number of equivalent functional metabolism pathways. In order to justify this idea, we proceed to explore and quantify the variety of mechanisms underlying the robustness inherent in each time scale. For this purpose, all the possible metabolic organization to reach the steady-state encrypted in the *modal* library was classified separately at each time scale as described in the next section.

### Aggregate variables: Pools of metabolites and their statistical variability

Experimental measurements on perturbed metabolic networks have revealed that some metabolites, called metabolic pools, move in a coordinated fashion along time scales [24]. Surprisingly, there is evidence that this temporal organization plays an important role in terms of establishing proper physiological conditions in cells [15], [18], [24]. From a mathematically perspective, these metabolic pools are defined by the row vectors along the *modal* library in such a way that first row of a modal matrix identifies the metabolic pools at the first time scale; the second row is associated with the second time scale, and so on. Continuing with our analysis, the *modal* library was statistically analyzed to survey the metabolic organization by which the cells respond to changes in kinetic parameters. The central aim is to survey the extent to which each metabolite participates in defining metabolic pools and whether some metabolites participate to an equal extent in spite of the selected kinetic parameters. Behind this analysis, I have hypothesized that the metabolites having a robust participation on metabolic pools can be potentially assigned certain fundamental physiological role. With this in mind, we defined two matrices (***M_ave_*** and ***N***) supplying information referring to the average and the dispersion for each entry along the entire *modal* library, see Figure 1.

Entries of these matrices *M_i,j_*(***N_i,j_***) identify the average (dispersion) contribution that the *j-th* metabolite have to define the metabolic pools at *i-th* time scale, see *methods section*. For instances the entry *M* ^3,1^_ave_ represents the average contribution of glucose 6 phosphate (g6p) at the third time scale, see Figure 2B. A similar explanation is given for the dispersion matrix ***N***.

Visual inspection of ***M_ave_*** reveals the formation of physiological metabolic pools emerging at slow time scales, see Figure 2C [15], [18]. Particularly, one can observe that at slow time scales the glucose 6-phosphate and the fructose 6-phosphate equally contribute to metabolic pools such that they move in a coordinated fashion starting from the 24^nd^ time scale. It indicates that at that time scale the reaction g6p = f6p driven by glucose 6-phosphate isomerase is at thermodynamic equilibrium. Equivalently, other metabolic pools suggesting an organized movement on its metabolite concentration were identified at subsequently time scales see Figure 2B bottom panel.

**Figure 4:**
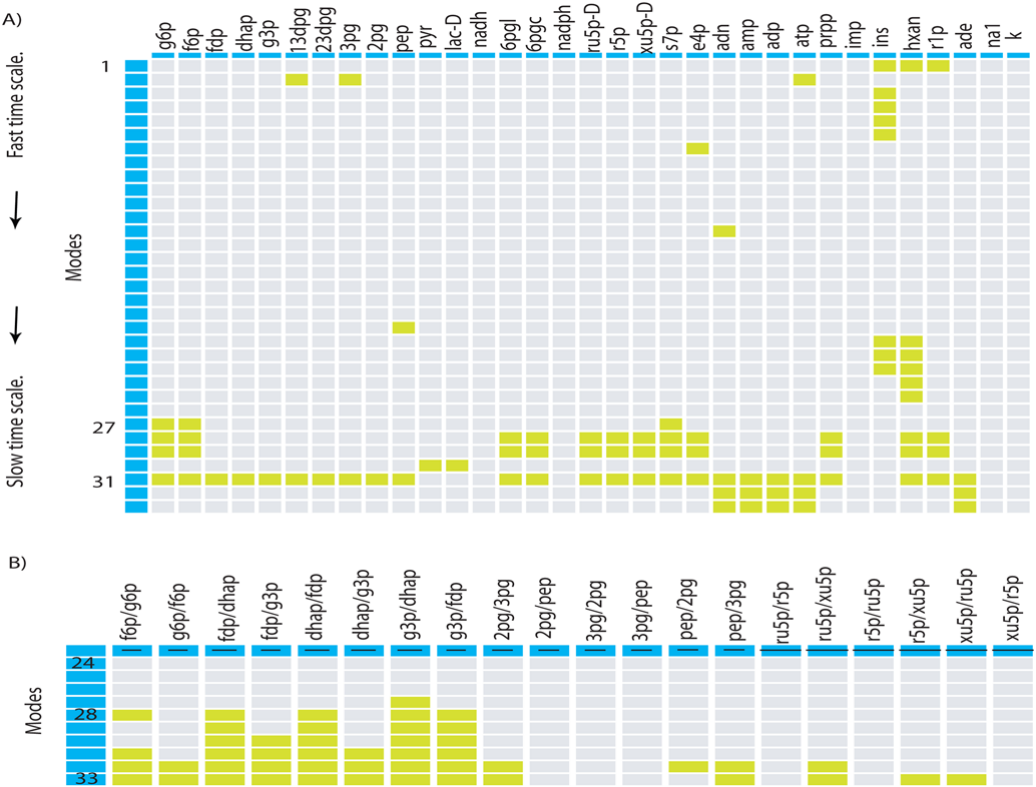
Dispersion in modal matrix. (A) Dispersion modal matrix shows those metabolites whose participation is robust to changes in kinetic parameter. In green-yellow we denote the metabolites whose dispersion was less than 1. B) Coefficients of variation obtained for some ratio of metabolites. As Figure shows, metabolites with robust participation in metabolic pools can be potentially identified at slow time scales. As in (A), these appear mainly at slow time scales.

On the other hand, Figures 4A shows the resulting ***N*** matrix. As previous interpretation, *i-th* row represents the dispersion of the metabolic pool formed at the *i-th* time scales and the columns quantify the dispersion of each metabolite. Figure 4A highlights in green-yellow the metabolites whose dispersion was lower than the unit, they assigned as potential metabolites that invariantly participates in metabolic pools independent of the used kinetic parameters. In addition, a statistical analysis accomplished for rates between some of these reveals the presence of additional robust properties, see Figure 4B. Based in these results one concludes that the organization of some metabolites to responds under changes in kinetic parameters tends to be robust at slow time scales.

### Redundancy on *modal* library

Robustness in metabolic networks has been attributed to a set of factors, with redundancy representing one of the most recognized [36]. In order to verify whether a kind of redundancy exist in *modal* library, we classify all the *i-th* rows along each matrix in *modal* library to construct a new sub-space called the *i-th metabolic pool* library. This sub-space have special relevance due that contains informative data referring to the degree of variation of metabolic pools in *modal* library. For example, the *3^rd^ metabolic pool* library is conformed by the collection of all metabolic pools identified at the *3^rd^* time scale along all *modal* library, so if redundancy does exist it should be hidden in that subspace. This classification allows us to construct 33 subspaces, each one consisting of 12586 rows corresponding to each time scale identified in previous section see Figure 1D.

**Figure 5:**
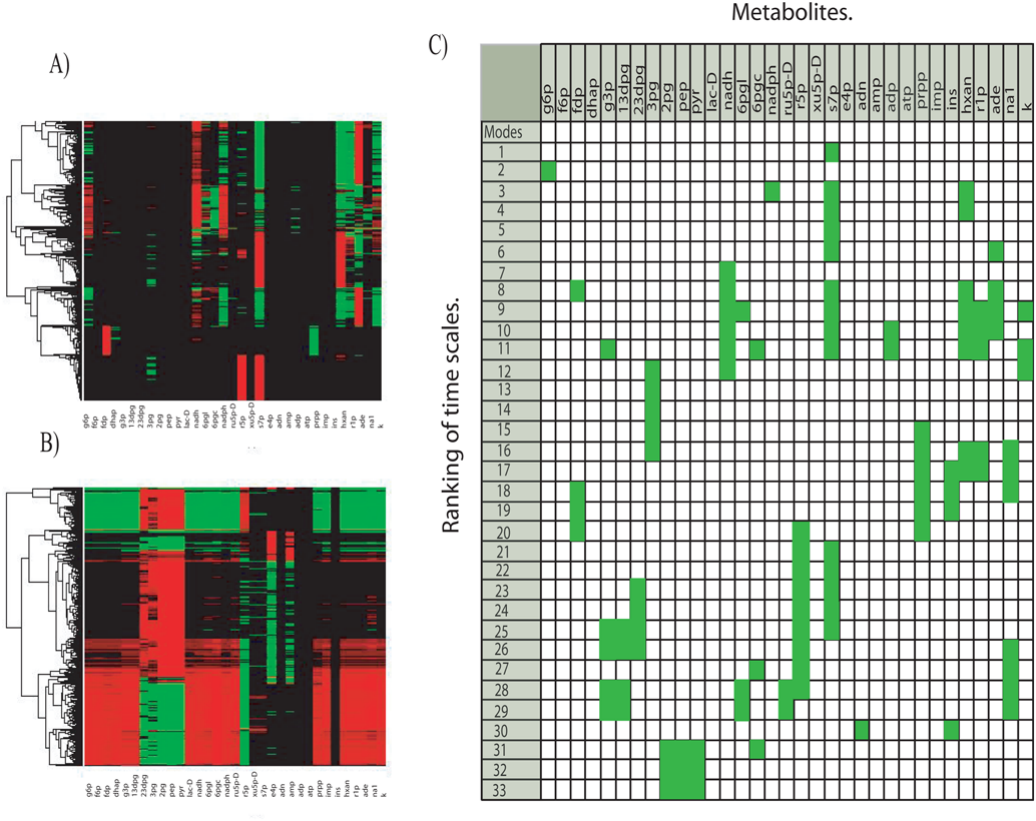
Redundancy in *modal* library. Figure (A) and (B) show a hierarchical cluster applied for the 6^nd^ and 32^nd^ *th-metabolic pool* library respectively. The presence (absence) of metabolites along the library is immediately observed by green/red (black) regions. From this Figure, metabolites with a redundant participation along the pools are identified. The degree of participation for each metabolite in each *i-th metabolic pool* library is depicted in (C). Green squares show the metabolites that participate at least 80% over the entire *th-metabolic pool* library.

Having performed this classification, we studied the metabolic composition for each *i-th metabolic pool* library by applying a hierarchical cluster analysis, whose visual representation reveals the degree of metabolite participation, redundancy and (dis)similarily between them. For instance, Figure 5A and 5B show the cluster analysis accomplished for the 6^th^ and 32^nd^ *metabolic pool* library respectively. In both cases, this graphical representation allows to identify conservative patterns through the space. Thus, for the 6^th^ *metabolic pool* library, we note that sedoheptulose 7-phosphate (r7p) participates over the pool formation in most of the situations. The inverse situation occurs with D-glucose 6-phosphate (g6p), fructose-6-phosphate (f6p), Fructose-1,6-diphosphate (fdp), dihydroxyacetone phosphate (dhap) and glyceraldehyde 3-phosphate (g3p) whose participation in metabolic pools would appear to be a function of kinetic parameters. Similarly, pyruvate (pyr), 2 phosphoglycerate (2pg) and phosphoenolpyruvate (pep) represent the predominant metabolites participating in the 32^nd^ *metabolic pool* library.

From a global analysis accomplished over the 33 *metabolic pool* libraries, one note that metabolites participation vary depending of the selected subspace. However, a frequency study accomplished over each *metabolic pool* library allows us to identify the potential metabolites that invariantly participate without reference on its kinetic parameters. Proceeding as described in methods section, for each *i-th metabolic pool* library we identify those metabolites that have contribute with at least with 80% over all the cases, the result is shown in Figure 5C. Overall, Figure 5 reveals a redundant pattern on the metabolic profile obtained form each *i-th metabolic pool* library. For these metabolites, redundancies may postulate them as key components to induce a proper physiological function, a hypothesis that should be experimentally verified through metabolome technology [26], [27].

### Variability of modal library

**Figure 6:**
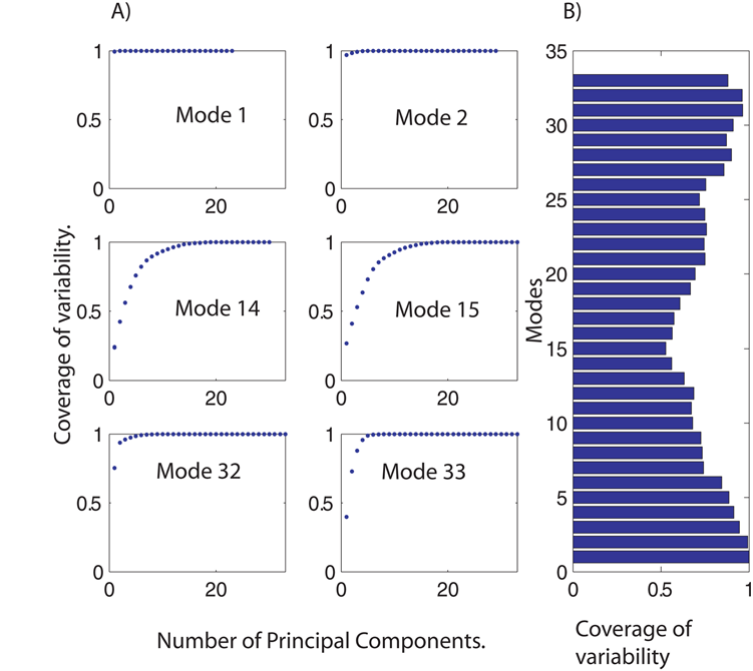
Variability in modal library. In order to quantify the variability on *i-th metabolic pool* library, Figure 1D, Principal Component Analysis was accomplished. Figure (A) shows the cumulative fraction of the complete space as a function of the number of principal components included in the description. As one appreciates the complete statistical behavior for all the libraries can be reproduced by a few numbers of principal components. Figure (B) depicts the variability fraction of the data covered by the five main principal components along the 33 *metabolic pool* spaces. The range of coverage goes from 72% to 100%.

Previous section has revealed valuable information concerning the redundant participation of metabolites along each *i-th metabolic pool* library, however it is needed to know how (dis)similar are the elements conforming each *i-th metabolic pool* library in order to have a better appreciation of the variability along the entire space. In order to quantify how similar are the elements of each *i-th metabolic pool* library, we applied Principal Component Analysis (PCA) [37]. Given a set of random variables, the central idea behind PCA is to calculate the minimal number of independent vectors, called principal components, required to reproduce the entire statistical properties prevailing in the original data set. Having applying PCA to each *i-th metabolic pool* library, one concludes that the number of independent variables present in the space can be accurately represented by a reduced set of principal components. In fact, the first five principal components are capable to reproduce between the 72%–100% of the complete variability along the *metabolic pool* libraries, see Figure 6. Detailed information concerning the percentage of *i-th metabolic pool* coverage achieved by the first five principal components for all the 33 libraries of metabolic pools is presented in Figure 6B.

In summary, along these last sections two observations stand out. Primarily, we have identified some metabolites that play part metabolic pools without reference to of kinetic parameters, this indicating a degree of redundancy of metabolic mechanisms to recover the steady-state condition, see Figure 5C. Secondly, statistical properties in terms of metabolic pools spaces can be largely be recovered (72%–100%) using five principal components indicating the reduced global variability nature for all the *i-th metabolic pool* libraries, see Figure 6.

## Discussion

The advent of high throughput technology and the increasing number of reconstructed metabolic networks constitutes essential components that have encouraged the development of *in silico* procedures capable of explaining how the metabolism in cell responds to external perturbations. Despite significant advances on dynamic theory [33], their practical counterpart to model genetic and metabolic circuits have been reported for some cases where kinetic parameters are experimentally known [38]. In order to analyze the physiological capabilities inherently associated with genome scale metabolic reconstructions, computational approaches capable to overcome this lacking of kinetic information are required. In this work, we have presented a new methodology for discovering dynamical mechanisms induced by a small perturbation in a metabolic network, where kinetic information is lacking. The workflow inherent to this method naturally integrates data from high throughput metabolome technology, in order to define the feasible space of kinetic parameters, the *k-cone*. Instead of following a deterministic description with well defined kinetic parameters, we have opted to consider a statistical procedure which leads to the construction of dynamic libraries for quantifying how and how fast the metabolic networks reached its steady-state, see Figure 1. This procedure can easily be applied to genome scale metabolic reconstructions and, as an example, we have applied it to study the human red blood cell metabolism. In this case, the range of time scales describing how and how fast the network reaches its steady-state were in agreement with those estimated using a detailed kinetic model [15]. This result makes possible the estimation of time scales associated with other available metabolic reconstructions, using their metabolome data and without necessarily requiring a complete knowledge of kinetic parameters. Furthermore, our statistical methodology can contribute to reveal the presence of robustness properties on metabolism.

As this paper shows, robustness, redundancy and variability were properties identified at different stages in perturbed metabolisms, all of them combined to give the global phenomenological effect. In general we conclude that robustness in time scale is caused by two types of metabolites: redundant and context dependent. The first classification confers redundancy to metabolic pools, and they are conformed by metabolites that always participate on the mechanics governing the relaxation toward a steady-state independently of the selected kinetic parameters. Conversely, context dependent metabolites are sensible to kinetic parameters and they supply variability to regulate the activity metabolites required to select the specific answer depending on the environment. From a systems biology perspective, there is evidence that different sort of robustness emerges inherently with network complexity [35], in this paper we report evidence of temporal robustness obtained from a genome scale metabolic analysis.

Overall, our method constitutes a framework for exploring dynamic behavior of slightly perturbed metabolic networks, where precise knowledge of kinetic information is lacking. Contrasting with others approaches, this method has been constructed such that data from high throughput metabolome technology [27] and genome scale metabolic reconstructions are fundamental elements to establish the integrative task between top-down and bottom up schemes [1].

Finally, I expect that this methodology can provide guidance in a future for exploring the relationship between dynamic and physiological behavior on other metabolic reconstructions. For instances, we envisage that it will contribute to design therapeutic targets in areas such as pharmacogenomics and genome medicine [36], [39], [40], where the estimation of feasible time scale and the identification of metabolic pools may be crucial for defining (dis)functional states. Particularly, exploring the relationship between high throughput technology and dynamic behavior on metabolic pathways directly associated with human diseases will thus constitute a mayor research target in future.

## Materials and Methods

### Human Red Blood Cell Metabolic Network

The metabolic network of Human Red Blood Cell (hRBC) analyzed in this paper is integrated by 33 metabolites participating in 68 internal and exchange reactions. These reactions integrate glycolysis, pentose phosphate pathway, Rapoport-Liubering and the nucleotides metabolism pathways. A graphical representation of this metabolic network can be found elsewhere [15], [30] and a more detail description of its properties is given in *Table S1* and *Table S2* in *supporting information*. In order to develop comparative studies and validate our predictions we use the same independent metabolites as reported in previous works [15], [18]. Metabolite concentrations defining the steady-state were estimated from the same sources [18]. A detailed description of the biochemical reactions, metabolites nomenclature and parameters used along the analysis is included in *Table S1* and *Table S2* at *supporting information*.

### *k-cone* formalism

*K-cone* analysis is a useful framework for estimating potential candidates of kinetic parameters underlying a metabolic network and ensuring the existence of a steady-state solution [29], [41]. Briefly, given a metabolic network with *m* metabolites and *n* reactions governed by law of mass action, the set of kinetic parameters (*k*) that ensure a steady state behavior are defined by
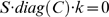
(1)Where *S* is the stoichiometric matrix, diag(.) represents a diagonal matrix and *C* is a 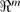 vector whose *i-th* entry is the product of the reactant concentration at steady-state corresponding to the *i-th* reaction, i.e.
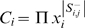
(2)Here *S^−^_i,j_* denotes the stoichiometric coefficients of reactants in the *j-th* reaction, in addition *x_i_* represents the known steady-state concentration for the *i-th* metabolite participating as reactant in the *j-th* reaction. In order to compare the results obtained with the statistical approach presented in this paper with the reported obtained from a detail kinetic model, we have used the same metabolite concentration at the steady-state as in previous reports [18], *see Table S1* in *supporting information*. Given that in general the number of reactions is greater that the number of metabolites, the kinetic solution of Equation 2 is determined by a myriad of points inside the null space of the matrix
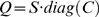
(3)Solution space forms a cone in a multidimensional space that integrate all the kinetic parameters potentially describing the biochemical network [29].

### Monte Carlo sampling of *k-cone* space

The identification of kinetic parameters integrating the *k-cone* was accomplished by an Artificial Center Hit and Run Algorithm (ACHR) [32], [42]. Basically, this algorithm defines an initial point along the null space of ***Q***. Once defined this point the algorithm calculate “warm-up” points from this initial point by an iterative procedure. These warm-up points are stored in a matrix **W** by which a centroid **x_c_** is calculated. Finally the sample points are calculated by selecting one point **y_n_** in the **W** matrix and moving in the direction vector given by (**x_c_ - y**). The new vector **y_n+1_** is substitute by the previous point **y_n_** in **W**. The centroid is recalculated and this process continues iteratively until a desired number of sample points are reached. ACHR was done using the COBRA tool box [43] selecting 19000 randomly distributed points with 1000 iterations between each sampled point. For the purpose of ensuring that dynamical behavior temporally converges to a steady-state, those kinetic parameters producing at least one positive eigenvalue from the *Jacobian* matrix were neglected. Overall, from the 19,000 sampled points in *k-cone* only 12586 were considered for all the statistic analysis.

### Dynamic analysis: Time Scales and Metabolic Pools

Linear perturbation theory considers the eigenvalues of the Jacobian matrix (λ*_i_*) as the informative parameters to estimate how fast a perturbed dynamical system reaches its equilibrium state [33], [44]. Thus, given a *Jacobian* matrix with rank *m*, one can deduce *m* time scales, τ*_i_*, calculated by τ*_i_* = −1/λ_i_ , *i* = 1..*m*. Thus, *hierarchical time scale* library was reconstructed by consecutively calculating the eigenvalue distribution for all matrices integrating the *Jacobian* library. For each realization, we ranked the eigenvalues, going from higher to lower magnitudes (faster to slower time scales) and the corresponding time scale was calculated. Average and standard deviation for the eigenvalues and the time scales along the entire distribution were calculated.

On the other hand, in order to explore how the metabolite relaxation occurs toward steady-state, a similarity transformation was applied to *Jacobian* matrix ***J***. This subtle transformation allows to write the original systems of coupled differential equation into a set of uncoupled differential equations whose new variables identify groups of metabolites that coordinately moves at each time scales, it means
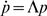
(4)With
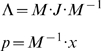
(5)Where Λ is a diagonal matrix whose entries are the eigenvalues of ***J***, ***M^−1^*** is the modal matrix and *p* is a vector defining the *metabolic pools*. Software implementation to obtain the time scale and the modal matrix was accomplished using *Mathematica 5.2*.

### Statistical analysis of modal matrices

Based on the *Jacobian* library and Equation 5, the *modal* library was obtained by storing 12586 different modal matrices. Consequently, a normalization procedure was implemented in each modal matrix by dividing in each row by the higher numerical absolute value. Having normalized, the average and the dispersion of the distribution obtained for each entry *M_i,j_* were obtained through Equation 6
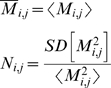
(6)Where *M_i,j_* and *N_i,j_* are components of the average and the dispersion modal matrix respectively. Brackets and SD[..] denotes average and standard deviation over the *modal* library respectively.

### Redundancy on *modal* library

In order to verify whether a kind of redundancy exist in *modal* library, we classify all the *i-th* rows along each matrix in *modal* library to construct a new sub-space called the *i-th metabolic pool* library. This sub-space have special relevance due that contains informative data referring to the degree of variation of metabolic pools in *modal* library. Thus, in order to discover the redundant participation of metabolites in metabolic pools, we proposed a function proper to this aim. Lets denote by 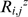 as the *j-th* metabolite contributing to the *i-th* row at the *z-th metabolic pool* library, see main text and Figure 1. Then, we defined a coefficient of participation quantifying the contribution that *j-th* metabolite have on the *z-th* metabolic pools as
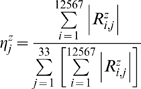
(7)Where |..| denotes the absolute value and *z* = 1,2,..33. This function essentially represents a normalized frequency of metabolite participation. The metabolites highlighted in Figure 5C are such that its coefficient of participation in *i-th metabolic pool* library is higher than 80% of the cases, *η^k^_j_*>0.8.

### Principal Component Analysis

With the aim to explore the degree of variability in *modal* library, Principal component analysis, PCA [37], was applied over each one of the 33 *i-th metabolic pool* libraries. As described in text, each subset *i-th metabolic pool* library was constructed by gathering all the modes identified at *i-th* time scale along the entire *modal* library. Minimal set of principal components was selected as five, it was justified considering that they were enough to reproduce between 72%–100% of the statistical variability presented along the 33 *modal* libraries, see Figure 6, right panel.

## Supporting Information

Figure S1 Time scale distribution for Human Red Blood Cell metabolism. Figure (A) shows the average time scales obtained from the statistical analysis. The coefficient of variation associated with each time scale is depicted in (B).
(1.06 MB EPS)

Table S1 Metabolite concentrations. Metabolite concentrations at the selected steady state condition. These data were estimated from references [15] and [18].
(0.21 MB RTF)

Table S2 Biochemical Network for Red Blood Cell Metabolism. Biochemical reactions included in the Red Blood Cell metabolic network.
(0.28 MB RTF)

Table S3 Range of Kinetic parameters. Range of the kinetic parameters obtained from k-cone space.
(0.30 MB RTF)
