# Supplementary material for: Filling Kinetic Gaps: Dynamic Modeling of Metabolism Where Detailed Kinetic Information Is Lacking
Source: PLoS One. 2009 Mar 23;4(3):e4967. doi: 10.1371/journal.pone.0004967 (PMC2654918; doi:10.1371/journal.pone.0004967)
Supplement: Supplementary file 1 [file pone.0004967.s002.rtf]

Abbreviation	Name	Steady-state concentration (mM)
13dpg	3-phospho-D-glyceroyl phosphate	2.28927 E-4
23dpg	2,3-Diphospho-D-glycerate	4.6545
2pg	D-glycerate 2-phosphate	0.0123
3pg	3-phospho-D-glycerate	0.0839
6pgc	6-phospho-D-gluconate	0.0375
6pgl	6-phospho-D-glucono-1,5-lactone	0.0018
ade	Adenine	0.001
adn	Adenosine	5.1923 E-5
adp	ADP	0.4902
amp	AMP	0.1488
atp	ATP	2.4016
dhap	Dihydroxyacetone phosphate	0.1474
e4p	D-erythrose 4-phosphate	0.0051
f6p	D-fructose 6-phosphate	0.0272
fdp	D-fructose 1,6-bisphosphate	0.0139
g3p	Glyceraldehyde 3-phosphate	6.7 E-3
g6p	D-Glucose 6-phosphate	0.0664
hxan	Hypoxanthine	2.2604 E-4
imp	IMP	1.92849 E-2
ins	Inosine	4.27616 E-5
k	Potasium	139.882
lac-D	Lactate	1.39709
na1	Sodium	14.1352
nadh	Nicotinamide adenine dinucleotide phosphate	3.059 E-2
nadph	Nicotinamide adenine dinucleotide phosphate	0.06559
pep	Phosphoenolpyruvate	0.0208
prpp	5-Phospho-alpha-D-ribose 1-diphosphate	0.0136
pyr	Pyruvate	0.06
r1p	alpha-D-Ribose 1-phosphate	0.0011
ru5p-D	D-ribulose 5-phosphate	4.93 E-3
s7p	Sedoheptulose 7-phosphate	2.39 E-2
xu5p-D	D-Xylulose 5-phosphate	1.47 E2
