# Supplementary material for: Filling Kinetic Gaps: Dynamic Modeling of Metabolism Where Detailed Kinetic Information Is Lacking
Source: PLoS One. 2009 Mar 23;4(3):e4967. doi: 10.1371/journal.pone.0004967 (PMC2654918; doi:10.1371/journal.pone.0004967)
Supplement: Supplementary file 2 [file pone.0004967.s003.rtf]

Abbreviation	Reaction	Name
**Glycolysis**		
HEX1	glc + atp -> adp + g6p	Glucokinase
PGI	g6p <=> f6p	Glucose-6-phosphate isomerase
PFK	atp + f6p -> adp + fdp	Phosphofructokinase
TPI	dhap <=> g3p	triosephosphate isomerase
GAPD	g3p + pi +nad <=> 13dpg + nadh + h	Glyceraldehyde3 phosphate dehydrogenase
PGK	3pg + atp <=> 13dpg + adp	phosphoglycerate kinase
DPGM	13dpg -> 23dpg	Phosphoglycerate mutase
DPGase	23dpg -> 3pg + p	Diphosphoglycerate phosphatase
PGM	2pg <=> 3pg	Phosphoglycerate mutase
ENO	2pg <=> pep	enolase
PYK	adp + pep -> atp + pyr	pyruvate kinase
LDHD	lac-D + nad <=> nadh + pyr	Lactate dehydrogenase
TALA	g3p + s7p < = > e4p + f6p	Transaldolase
FBA	fdp < = >dhap + g3p	fructose biphosphate aldolase
ATPase	atp -> adp	ATPase
**Pentose phoshate pathway**		
G6PDH2	g6p -> 6pgl + nadph	glucose-6-phosphate dehydrogenase
PGL	6pgl - > 6pgc	6-phosphoglyconolactonate
GND	6pgc -> nadph + ru5p-D	6-phosphoglycononate dehydrogenase
RPI	r5p < = > ru5p-D[c]	Ribulose 5-phosphate isomerase
RPE	ru5p-D < = > xu5p-D	Ribulose 5-phosphate 3-epimerase
TKT1	r5p + xu5p-D <=> g3p + s7p	Transketolase
TKT2	e4p + xu5p-D <=> f6p + g3p	Transketolase
**Adenosine Nucleotide Metabolism**		
ADA	adn -> ins	Adenosine deaminase
ADNK1	adn + atp -> adp + amp	Adenosine kinase
ADK1	2 adp < = > atp + amp	Adenylate kinase
AMPDA	amp -> imp	Adenosine monophosphate phosphohydrolase
ADPT	ade + prpp -> amp	Adenine phosphoribosyl transferase
NTD11	imp -> ins	inosine monophosphatase
PUNP5	ins < = > hxan + r1p	purine nucleoside phosphorylase
PPM	r1p < = > r5p	phosphoribomutase
PRPPS	atp + r5p - > amp + prpp	phosphoribosyl pyrophosphate synthetase
HXPRT	hxan + prpp - > imp +2 p	Hypoxanthine guanine phosphoryl transferase
ATPM	atp < = = adp + p	ATPase/ATP maintenance requirement
**Exchange and demand reactions**		
NaKt	atp + 3 na1 - >adp + 2 k	Na+/ k+ exchange ATPase
PYRt2r	pyr < = >	pyruvate exchange
D-LACt2	lac-D < = >	lactate exchange
HXANt	hxan - >	Hypoxanthine exchange
INSt	ins < = >	Inosine exchange
ADEt	ade < = >	Adenosine exchange
ADNt	-> adn	Demand reaction.
NADH	nadh < = >	NADH exchange
NADPH	nadph - >	nadph exchange
DPG23	23dpg - >	23dpg exchange
NAt	na1 < = >	Sodium exchange
Kt1	k < = >	Potasium exchange
