# Supplementary material for: Filling Kinetic Gaps: Dynamic Modeling of Metabolism Where Detailed Kinetic Information Is Lacking
Source: PLoS One. 2009 Mar 23;4(3):e4967. doi: 10.1371/journal.pone.0004967 (PMC2654918; doi:10.1371/journal.pone.0004967)
Supplement: Supplementary file 3 [file pone.0004967.s004.rtf]

Numerical label	Reaction	Kmin	Kmax	Numerical label	Reaction	Kmin	Kmax
1	sink_nadh(c)	3.6642	3268.04	35	NAt_f	0.372	99.9
2	sink_nadh(c)_b	0.00238	99.7086	36	NAt_b	1.0212798 E-6	7.0121
3	sink_nadph(c)_f	0.0037	177.7295	37	NTD11	0.7456	2339.5614
4	ADA	0.0281	2866.979	38	NaKt	1.635708 E-8	0.0048
5	ADEt_f	0.0002063	0.099	39	PFK	0.0024	30.2
6	ADEt_b	0.00435	96.04376	40	PGI_f	0.017	1504.9
7	ADK1_f	1.8661	279.77	41	PGI_b	0.345	3671.79
8	ADK1_b	0.0182	415.4368	42	PGK_f	0.98	496.11
9	ADNK1	0.01908	1196.607	43	PGK_b	81.358	890152.92
10	ADNt_f	0.00033	0.149	44	PGK_b	0.18353	3344.6753
11	ADPT	0.0207	7051.176	45	PGL	0.20652501	8140.6402
12	AMPDA	0.00703	1.5607	46	PGM_f	0.35526002	1191.4246
13	ATPM	0.00169	40.22	47	PGM_b	188.26364	85234.611
14	D-LACt2_f	0.000051	2.59	48	PPM_f	0.0441	7854.8775
15	D-LACt2_b	0.00034	1.861	49	PPM_b	0.36269829	1435.4059
16	DPGM_f	457.48623	372594	50	PUNP5_f	6982.5037	2.33 E6
17	DPGase	0.00015	18.158	51	PUNP5_b	25857.78	3.77 E8
18	ENO_f	5.379	8151.004	52	PYK	0.01245	340.296
19	ENO_b	0.0707	4783.551	53	PYRt2r_f	0.0000613	2.5999667
20	FBA_f	9.028	7213.589	54	PYRt2r_b	0.00198	43.331
21	FBA_b	5.595	101254.8	55	RPE_f	22.95	20254.534
22	G6PDH2	0.00184	87.739	56	RPE_b	1.094	6761.358
23	GAPD_f	107.981	14928.53	57	RPI_f	0.119	7884.32
24	GAPD_b	828.116	1.416 E7	58	RPI_b	40.995	20255.893
25	GND	0.0032	155.553	59	TALA_f	901.4684	622327.23
26	HEX1	0.0392	0.8327	60	TALA_b	7.78956	723054.88
27	HXANt2	2.951 E-6	0.099	61	TEST_ATPASE	9.811	203.9931
28	HXPRT	170.934	1.4590412	62	TKT1_f	348.1627	533884.1
29	INSt_f	0.000021	0.697	63	TKT1_b	75.806	621863.7
30	INSt_b	1340.664	17305.17	64	TKT2_f	1697.402	1.332 E6
31	Kt1r_f	0.00142	99.631	65	TKT2_b	25.794	547901.19
32	Kt1r_b	0.0064	0.7148	66	TPI_f	0.279	678.30944
33	LDH_D_f	0.00106	71.56782	67	TPI_b	0.82214831	14904.6
34	LDH_D_b	10.799	54466.73	68	Ex_23dpg	0.000032	0.64448
